# Feature separation and adversarial training for the patient-independent detection of epileptic seizures

**DOI:** 10.3389/fncom.2023.1195334

**Published:** 2023-07-19

**Authors:** Yong Yang, Feng Li, Xiaolin Qin, Han Wen, Xiaoguang Lin, Dong Huang

**Affiliations:** ^1^Chengdu Institute of Computer Application, Chinese Academy of Sciences, Chengdu, Sichuan, China; ^2^Chongqing Institute of Green and Intelligent Technology, Chinese Academy of Sciences, Chongqing, China; ^3^Chongqing School, University of Chinese Academy of Sciences, Chongqing, China; ^4^Department of Neurology, The First Affiliated Hospital of Chongqing Medical University, Chongqing, China

**Keywords:** epileptic seizure detection, EEG, feature separation, adversarial training, patient-independent

## Abstract

An epileptic seizure is the external manifestation of abnormal neuronal discharges, which seriously affecting physical health. The pathogenesis of epilepsy is complex, and the types of epileptic seizures are diverse, resulting in significant variation in epileptic seizure data between subjects. If we feed epilepsy data from multiple patients directly into the model for training, it will lead to underfitting of the model. To overcome this problem, we propose a robust epileptic seizure detection model that effectively learns from multiple patients while eliminating the negative impact of the data distribution shift between patients. The model adopts a multi-level temporal-spectral feature extraction network to achieve feature extraction, a feature separation network to separate features into category-related and patient-related components, and an invariant feature extraction network to extract essential feature information related to categories. The proposed model is evaluated on the TUH dataset using leave-one-out cross-validation and achieves an average accuracy of 85.7%. The experimental results show that the proposed model is superior to the related literature and provides a valuable reference for the clinical application of epilepsy detection.

## 1. Introduction

Epilepsy is a chronic disorder caused by the sudden abnormal discharge of nerve cells in the brain, resulting in temporary brain dysfunction. Epilepsy is the second most common neurological disorder after headache, affecting approximately 70 million people worldwide. The clinical manifestations of epileptic seizures are complex, and the types of epileptic seizures are varied. The clinical manifestations may include impaired consciousness, limb spasms, urinary incontinence, frothing, and other symptoms. Although epileptic seizures have little impact on patients in the short term, long-term frequent seizures have a severe impact on the physical, mental, intellectual health of patients (Rakhade and Jensen, 2009; Rasheed et al., 2021). Most people with epilepsy can control their condition with medication and surgery, still, about 30% of people with intractable epilepsy cannot be adequately controlled with medication (Kwan and Brodie, 2000), posing a severe threat to the life and health of patients and a heavy burden to their families and society.

The pathogenesis of epilepsy is complex, and the types of epileptic seizures are varied. The characteristics of EEG (electroencephalogram) data during the epileptic seizure period are related to the original location and cause of epilepsy. Different diseases of the nervous system or various conditions of the brain can cause different epileptic seizures, and the same condition of the nervous system can cause more than one type of epileptic seizure. Previous studies have pointed out that about 7% of the neurons ignited in patients with subclinical seizure, about 14% of the neurons ignited in patients when omen appeared. About 36% of the neurons ignited in patients with clinical seizure. Therefore, in the same patient, the intensity, type, location, duration of each seizure may be the same or different. In multiple patients, the differences are more marked (Babb et al., 1987; Fisher et al., 2017).

Most of the existing epileptic seizure detection methods focus on the patient-dependent scenario, which refers to detecting a patient’s epileptic seizure by learning from his own historical records; this method is easy to implement and has high detection accuracy. In contrast, patient-independent methods advance in alerting potential patients but are easily corrupted by inter-patient noises. Most existing studies fail to eliminate significant differences between patients (mainly caused by multiple factors such as physical condition, pathogenesis, seizure intensity, seizure type, etc.). When the model is trained directly on data from multiple patients, it will easily lead to underfitting, and detection performance will drop sharply on new patients. For these reasons, we propose a new method, which uses a feature extraction network and feature separation network to improve the discriminability of features, and which uses the marginal distribution and conditional distribution alignment technology of features to enhance the ability to extract patient invariant features.

The main contributions of our study can be summarized as follows:

(1) We propose a novel domain generalization model based on feature disentanglement and adversarial training to enhance the ability of extracting patient invariant features, so the generalization ability of the model is improved.
(2) We verify the proposed model through extensive experimental evaluations. The experimental results show that our proposed approach has significant potential to provide an optimal epileptic seizure detection method, and it also provides a valuable reference for clinical application.

The remainder of this paper is organized as follows. In the section “2. Related work,” reviews the related work of epileptic seizure detection. In the section “3. Methodology,” a patient-independent epileptic seizure detection model is proposed. In the section “4. Experiments,” we present experiments and results on a benchmark dataset. In the section “5. Discussion,” we analyze the effectiveness of the proposed method. Finally, some conclusions are given in the section “6. Conclusion.”

## 2. Related work

As a subclass of machine learning, deep neural networks have made remarkable progress in computer vision, natural language processing, and other fields, and researchers have proposed a variety of network models and methods for specific application scenarios. In the research of domain generalization methods, the following two approaches are usually adopted: (1) The method based on experience and knowledge is designed to extract universal features that can perform good detection on new patients. (2) The domain adaptive technology is used to extract invariant features of multiple patients to improve the generalization ability of the model.

For the first approach, Ansari et al. (2021) proposed an automated seizure onset detection system, which used power spectrum features and some statistical features to detect seizure onset, achieving a mean latency of 0.9 s and 1.02 false detections per hour. Liu et al. (2022) proposed a novel patient-independent approach; this method used wavelet decomposition, Convolutional Neural Network (CNN), Bidirectional Long Short-Term Memory (Bi-LSTM) network and a novel channel perturbation technique, achieved mean accuracies of 97.51 and 93.70%. Sridevi et al. (2019) proposed a patient-independent approach; this method used spectral entropy, spectral energy and signal energy as useful features, achieved a better classification effect.

For the second approach, Zhao et al. (2021) proposed a domain adaptive method, domain shift can be eliminated from the source domain to the target domain, and achieved better performance. Li et al. (2021) proposed a bi-hemisphere domain adversarial neural network, that achieved good recognition performance in EEG emotion recognition. Tang and Zhang (2020) applied conditional adversarial domain adaptation neural network to motor image EEG decoding, and achieved a better classification effect.

In epilepsy detection, Zhang et al. (2020) used feature separation and adversarial representation learning methods to decompose the data into categories (seizure and normal) related features and patient-related features, achieving an average accuracy rate of 80.5% on the TUH EEG dataset. Dissanayake et al. (2021) used the CNN network structure and Siamese network structure, and achieved an accuracy of 88.81% on the CHB-MIT dataset.

To the best of our knowledge, the above methods do not completely eliminate the effects of the data distribution shift between patients, so in this study, we propose a robust approach to address this problem.

## 3. Methodology

### 3.1. The proposed network

**FIGURE 1:**
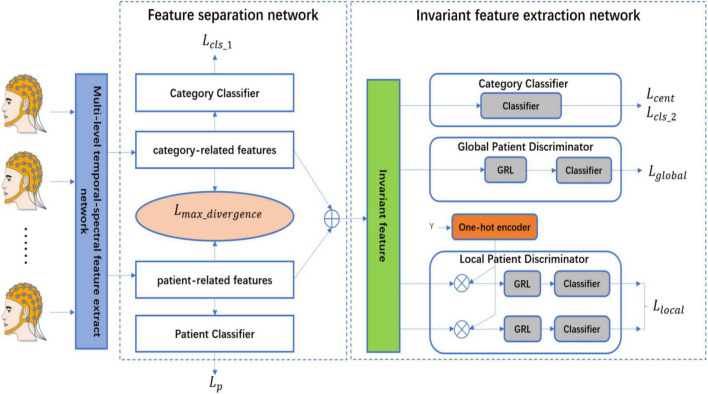
The architecture of the proposed network.

**FIGURE 2:**
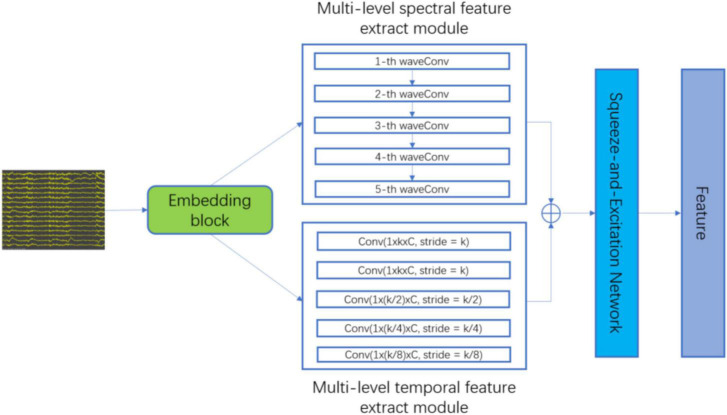
The architecture of multi-level temporal-spectral feature extract network.

The proposed patient-independent epileptic seizures detection model is illustrated in Figure 1, which includes three subnets. (1) Multi-level temporal-spectral feature extraction network, (2) feature separation network, and (3) invariant feature extraction network. The feature extraction network extracts temporal feature information and frequency domain feature information from EEG data (Li et al., 2020), and performs enhanced characterization by the Squeeze-and-Extraction Network (Hu et al., 2018), so that the extracted features are discriminable; the feature extraction network is illustrated in Figure 2. The feature separation network disentangles the features into category-related features and patient-related features. Finally, the invariant feature extraction network extracts the invariant patient-independent features by aligning the marginal distribution and the conditional distribution; so the generalization ability of the model is improved.

### 3.2. Multi-level temporal-spectral feature extract network

Electroencephalogram data is two-dimensional data similar to images, which has uncertainties and incidences; therefore, it is necessary to preprocess the original data; we use min-max regulation technology to regulate the data. You can also refer to Rahim et al. (2016) and Versaci and Morabito (2021) for preprocessing.

As convolution operators are essentially equivalent to a low-pass filter (Azimi et al., 2019), the embedding block, the embedding block, that is, successive temporal convolution and batch normalization (BN) operations, is first adopted to infer an optimal filter-band for the subsequent analysis. As a result, after stacking original data and output embeddings with a channel-wise concatenation function, the embedding block obtains a sub-band matrix, which provides a subsequent network with adaptive sub-band responses and also original data. Finally, the data is fed into the multi-level spectral feature extraction module and the multi-level temporary feature extraction module for feature extraction.

In the multi-level temporal-spectral feature extraction network, in order to prevent the deformation of the boundary data caused by zero padding in the convolution operation, the head and tail of the data are filled according to formula (1):


(1) $$x_{p}=x\left(N-\frac{R}{2}+1\right),\ldots ,x\left(N-1\right)|x\left(0\right),\ldots ,x\left(N-1\right)|x\left(0\right),\ldots ,x\left(\frac{R}{2}-2\right)$$

Where, | is a concatenating operator, *x*(*i*) is the *i*-th element of input *x*, *R* representing the parameter kernel size in the convolution operation.

In order to reduce the time of data computation, the proposed method adopts convolution operation to perform multi-level wavelet decomposition, which is defined as follows:


(2) $$y_{A}\,\left(i\right)=\left(x_{p}⊗g\right)\,\left(i\right)=\sum_{r=0}^{R}x_{p}\,\left(s\times i-r\right)\times g\,\left(r\right)$$

(3) $$y_{D}\,\left(i\right)=\left(x_{p}⊗h\right)\,\left(i\right)=\sum_{r=0}^{R}x_{p}\,\left(s\times i-r\right)\times h\,\left(r\right)$$

Where, ⊗ is the convolution operation, *g* and *h* represent a pair of scaling and wavelet filter, *s* represents the parameter stride in the convolution operation, *y*_*A*_(*i*) is the approximation (low pass) coefficients, and *y*_*D*_(*i*) is the detail (high pass) coefficients.

In the multi-level spectral feature extraction module, to extract the corresponding wavelet coefficients under standard physiological sub-bands δ(0∼4 Hz), θ(4∼8 Hz), α(8∼16 Hz), β(16∼32 Hz), and γ(32∼64Hz), we select Daubechies order-4 (Db4) wavelet, since previous studies reported that Db4 mother wavelet is useful for epileptiform transient detection due to its high correlation coefficients with the epileptic spike signal (Indiradevi et al., 2008). Finally, the frequency features (*f*_δ_, *f*_θ_, *f*_α_, *f*_β_, *f*_γ_) are obtained.

In the multi-level temporal feature extraction module, considering the data distribution shift between subjects, we use five independent convolution, batch normalization and empirical linear unit (ELU) operations to capture multi-level temporal feature information with different receptive fields. The convolution kernel size is set to [S, 1], the value of S is {*k*, *k*, *k*/2, *k*/4, *k*/8}, *k* = 2^5^, and finally, the temporary features (*f*_*t*1_,*f*_*t*2_,*f*_*t*3_,*f*_*t*4_,*f*_*t*5_) are obtained.

To further extract discriminative feature information, the features extracted by the multi-level spectral feature extraction module and the multi-level temporal feature extraction module are combined according to the feature dimensions:


(4) $$f_{a\,l\,l}=\left\{\left[f_{\delta }|f_{t\,1}\right],\left[f_{\theta }|f_{t\,2}\right],\left[f_{\alpha }|f_{t\,3}\right],\left[f_{\beta }|f_{t\,4}\right],\left[f_{\gamma }|f_{t\,5}\right]\right\}$$

The combined features *f*_*all*_ are fed into Squeeze-and-Excitation Network to enhance feature discrimination.

### 3.3. Feature separation network

The feature information (category information, patient information, etc.) is contained in each dimension and intertwined. If the features can be disentangled by the feature separation network, the separability and discriminability of the features will be improved. Therefore, according to the prior knowledge, we separate the features which are obtained from the feature extraction network into two parts, the first half of the features is the category-related component, which is recorded as *F*_*category*_*related*_, the second half of the features is the patient-related component, which is recorded as *F*_*patient*_*related*_. In addition, to ensure the first half of the features are the category-related component, the category classifier and cross-entropy loss function are used, to ensure the second half of the features are the patient-related component, the patient classifier and cross-entropy loss function are used, to ensure better separation of the features of the two parts, the maximum divergence loss function is used to ensure the maximum separation of the category-related component and the patient-related component (Bui et al., 2021).

The loss function of the category classifier and the patient classifier is:


(5) $$L_{c\,l\,s\,\_\,1}=\frac{1}{N}\,\sum_{x_{i}\in D_{s}}L\,\left(G_{c\,1}\,\left(G_{f}\,\left(x_{i}\right)\right),y_{i}\right)$$

(6) $$L_{p}=\frac{1}{N}\,\sum_{x_{i}\in D_{s}}L\,\left(G_{p}\,\left(G_{f}\,\left(x_{i}\right)\right),d_{i}\right)$$

Where, *N* is the number of samples, *x*_*i*_ is the data sample, *G*_*f*_ is the feature extraction network, *G*_*c*1_ is the category classifier, *G*_*p*_ is the patient classifier, *L* is the cross-entropy loss function, y_*i*_ is the category label (seizure or normal), *d*_*i*_ is the patient label, *D*_s_ ∈ *D*_1_ ∪ *D*_2_… ∪ *D*_n_ (*D*_1_,*D*_2_,……, *D*_*n*_ are the data of each patient).

To separate category-related component (*F*_*category*_*related*_) and patient-related component (*F*_*patient*_*related*_), we use the maximum divergence loss function:


(7) $$L_{m\,a\,x\,\_\,d\,i\,v\,e\,r\,g\,e\,n\,c\,e}=-\sum_{i=1}^{N}{\left(F_{c\,a\,t\,e\,g\,o\,r\,y\,\_\,r\,e\,l\,a\,t\,e\,d}-F_{p\,a\,t\,i\,e\,n\,t\,\_\,r\,e\,l\,a\,t\,e\,d}\right)}^{2}$$

Then combine the separated features to create new features:


(8) $$F_{a\,l\,l}^{\prime }=\text{[}F_{c\,a\,t\,e\,g\,o\,r\,y\,\_\,r\,e\,l\,a\,t\,e\,d}|F_{p\,a\,t\,i\,e\,n\,t\,\_\,r\,e\,l\,a\,t\,e\,d}]$$

### 3.4. Invariant feature extraction network

The feature separation network effectively disentangles the features and improves the discrimination of the features, but the current features are not the invariant features of each patient. To improve the generalization ability of the model, the proposed method is based on the methods of DANN (Domain-adversarial training of neural networks) (Ganin et al., 2016; Yu et al., 2019) and MADA (Multi-adversarial domain adaptation) (Pei et al., 2018) to achieve better invariant feature learning. The global patient discriminator aligns the features of each patient according to the marginal distribution. The local patient discriminator aligns the features of each category according to the conditional distribution. The global adversarial loss function and the local adversarial training loss function are as follows:


(9) $$L_{g\,l\,o\,b\,a\,l}=\frac{1}{N}\,\sum_{x_{i}\in D_{\text{s}}}L\,\left(G_{g}\,\left(G_{f}\,\left(x_{i}\right)\right),d_{i}\right)$$

(10) $$L_{l\,o\,c\,a\,l}=\frac{1}{N}\,\sum_{\text{k}=1}^{K}\sum_{x_{i}\in D_{\text{s}}}L\,\left(G_{l}^{k}\,\left(y_{i}^{k}\,G_{f}\,\left(x_{i}\right)\right),d_{i}\right)$$

Where, *L* is the cross entropy loss function, *G*_*f*_ is the feature extraction network, *G*_*g*_ and $G_{l}^{k}$ (*k* = 1,2) are the patient discrimination network, *d*_*i*_ is the patient label, $y_{i}^{k}$ (*k* = 1,2) is the first and second dimensional data of the original label after one-hot encoder, *D*_s_ ∈ *D*_1_ ∪ *D*_2_… ∪ *D*_n_ is the patient sample set.

In category classifier, to centralize the character of data, the central loss function is adopted. The loss function is (Wen et al., 2016):


(11) $$L_{c\,e\,n\,t}=\frac{1}{2}\,\sum_{i=1}^{M}{||x_{i}-c_{y_{i}}||}_{2}^{2}$$

Where, *c*_*y*_*i*__ is the category center.

Through the above operations, the marginal distribution and conditional distribution of features are aligned, and the features are gathered to the central point of each category, so the invariant features are obtained. The loss function of the category classifier (Rahim et al., 2015; White et al., 2020; Versaci et al., 2022; Waheed et al., 2023) is:


(12) $$L_{c\,l\,s\,\_\,2}=\frac{1}{N}\,\sum_{x_{i}\in D_{s}}L\,\left(G_{c\,2}\,\left(G_{f}\,\left(x_{i}\right)\right),y_{i}\right)$$

Where, *G*_*c*2_ is the category classifier, *y*_*i*_ is the category label.

### 3.5. Training details

We propose an adversarial training strategy to train all the loss functions jointly (Matsuura and Harada, 2020):


(13) $$L_{s\,u\,m}=L_{c\,l\,s\,2}+\lambda \times \left(L_{c\,e\,n\,t}+L_{c\,l\,s\,1}+L_{s\,u\,b\,j\,e\,c\,t}+L_{m\,a\,x\,\_\,d\,i\,v\,e\,r\,g\,e\,n\,c\,e}\right)$$

$$-\lambda \times \left(L_{g\,l\,o\,b\,a\,l}+L_{l\,o\,c\,a\,l}\right)$$

Where, λ = 0.1. $\theta_{g},\theta_{l}^{1},\theta_{l}^{2}$ are trained by a special layer called Gradient Reversal Layer (GRL), this GRL is omitted during forward propagation, and the gradient is reversed in backpropagation. Finally, we search for the optimal parameters θf∧f,θc⁢2∧c⁢2,θg∧g,θl1∧1,θl2∧2 to meet the following requirements:


(14) $$\left(\overset{\wedge }{\theta_{f}}\overset{\wedge }{\theta_{c2}})=\arg\,\min\underset{\theta_{f},\theta_{c2}}{L_{sum}}(\theta_{f},\theta_{c1},\theta_{p},\theta_{c2},\theta_{g},\theta_{l}^{1},\theta_{l}^{2}\right)$$

(15) $$\left(\overset{\wedge }{\theta_{g}},\overset{\wedge }{\theta_{l}^{1}},\overset{\wedge }{\theta_{l}^{2}})=\arg\,\max\underset{\theta_{g},\theta_{l}^{1},\theta_{l}^{2}}{L_{sum}}(\theta_{f},\theta_{c1},\theta_{p},\theta_{c2},\theta_{g},\theta_{l}^{1},\theta_{l}^{2}\right)$$

Where, θ_*f*_ are the parameters of multi-level temporal-spectral feature extract network, θ_*c*1_ are the parameters of category classifier in feature separation network, θ_*p*_ are the parameters of patient classifier in feature separation network, θ_*c*2_ are the parameters of category classifier in invariant feature extraction network, θ_*g*_ are the parameters of global patient discriminator in invariant feature extraction network, $\theta_{l}^{1},\theta_{l}^{2}$ are the parameters of local patient discriminator in invariant feature extraction network.

During training, if the training samples are trained by minibatch, the features of all the training samples cannot be obtained in time, so we feed all the training samples into the network as a batch for training. The Adam optimizer is used for the model; the learning rate is set to 0.005; the center loss function is optimized using the Stochastic Gradient Descent (SGD) optimizer, and the learning rate is set to 0.05; the training rounds are 200. We use the grid search method to set the hyperparameters in the experiment.

## 4. Experiments

### 4.1. Dataset

The proposed approach is evaluated on a benchmark dataset, the TUH corpus (Obeid and Picone, 2016), which is a neurological seizure dataset of clinical EEG recordings associated with 22 channels according to the international 10/20 system. We form a subset of the TUH with 14 subjects by selecting the subject with more than 250 s of seizure state. For each subject, we use 500 s (half normal and half seizure) of EEG signals with a sampling rate of 250 Hz. Each EEG fragment has 250 sample points (lasting 1 s) and adjacent fragments with 50% overlap. For each EEG fragment, those belonging to the epileptic seizure state are labeled as 1, while those belonging to the normal state are labeled as 0. Then the sample set is divided into a training set and a test set.

### 4.2. Evaluate metrics

The experiment used accuracy (ACC), sensitivity (SN), and specificity (SP) to quantify the performance of the algorithm (Yang et al., 2023).


(16) $$A\,C\,C=\frac{T\,P+T\,N}{T\,P+T\,N+F\,P+F\,N}$$

(17) $$S\,N=\frac{T\,P}{T\,P+F\,N}$$

(18) $$S\,P=\frac{T\,N}{T\,N+F\,P}$$

Where, TP (True Positive): The sample which is positive is judged to be positive, TN (True Negative): The sample which is negative is judged to be negative, FP (False Positive): The sample which is negative is judged to be positive, FN (False Negative): The sample which is positive is judged to be negative.

### 4.3. Baselines

The adopted baseline models include:

● Zabihi et al. (2013) applied Discrete Wavelet Transform (DWT) and calculated metrics such as relative scale energy and Shannon entropy as features; SVM is used for data classification.

● Fergus et al. (2015) applied Power Spectral Density (PSD) and calculated metrics such as peak frequency and max frequency as features; KNN is used for data classification.

● Schirrmeister et al. (2017) applied convolutional neural networks to distinguish seizure segments by decoding task-related information from EEG signals.

● Kiral et al. (2018) designed a deep neural network for seizure diagnosis and further developed a prediction system on a wearable device.

● Zhang et al. (2020) proposed an adversarial representation learning strategy, which achieves robust and explainable epileptic seizure detection.

● Dissanayake et al. (2021) used the CNN network structure and Siamese network structure to improve the generalization ability of the model.

The six comparison methods and my experiment used the same data segment length on the TUH dataset, using leave-one-out cross-validation, and obtained the comparison results in Table 1.

**TABLE 1:** Performance comparison on the TUH dataset.

References	Subject ID														
	**0**	**1**	**2**	**3**	**4**	**5**	**6**	**7**	**8**	**9**	**10**	**11**	**12**	**13**	**Average**
Zabihi et al., 2013	0.821	0.746	0.719	0.706	0.726	0.773	0.804	0.863	0.762	0.758	0.832	0.758	0.784	0.816	0.776
Fergus et al., 2015	0.803	0.775	0.865	0.751	0.801	0.718	0.853	0.898	0.722	0.758	0.866	0.725	0.791	0.823	0.796
Schirrmeister et al., 2017	0.793	0.743	0.965	0.758	0.789	0.665	0.813	0.871	0.619	0.634	0.919	0.571	0.744	0.711	0.760
Kiral et al., 2018	0.805	0.669	0.855	0.709	0.772	0.619	0.823	0.836	0.746	0.598	0.835	0.556	0.745	0.726	0.736
Zhang et al., 2020	0.841	0.826	0.978	0.774	0.842	0.733	0.911	0.914	0.697	0.652	0.923	0.604	0.772	0.787	0.805
Dissanayake et al., 2021	0.804	0.831	0.792	0.726	0.814	0.834	0.879	0.758	0.808	0.782	0.892	0.885	0.856	0.855	0.823
Ours	0.820	0.728	0.924	0.604	0.860	0.944	0.984	0.904	0.876	0.884	0.900	0.932	0.812	0.836	0.857

Through comparative analysis, the methods in literature (Schirrmeister et al., 2017; Kiral et al., 2018) only used a deep neural network to train a model with the data of multiple patients together, without considering the negative impact of inter-patient differences on the training model, resulting in poor detection accuracy when applied to new patients. In literature (Zabihi et al., 2013), relative scale energy and Shannon entropy, etc., were used as features, in literature (Fergus et al., 2015), peak frequency and max frequency, etc., were used as features, these methods were able to extract the obvious common features, but were unable to extract the deeper common features, so the detection accuracy of the methods was higher than the results in Schirrmeister et al. (2017) and Kiral et al. (2018) and lower than the results in Zhang et al. (2020) and Dissanayake et al. (2021). For the methods mentioned in the literature (Zhang et al., 2020; Dissanayake et al., 2021), which applied a neural network to eliminate the negative impact of the data distribution shift between patients, the results were higher than those without considering the elimination of the negative impact of the data distribution shift between patients. For the method proposed in this paper, which uses feature separation and adversarial training to disentangle features in the latent space while learning domain-invariant features to achieve the goal of mitigating the influence of inter-patient differences, its experimental results are the best, with an average detection accuracy of 85.7% by leave-one-out cross-validation.

**FIGURE 3:**
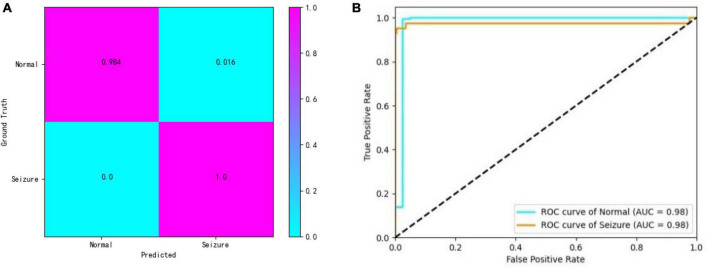
**(A,B)** Confusion matrix and ROC curves.

In addition, the confusion matrix and the receiver operating characteristic (ROC) curve with the area under the curve (AUC) value are shown for a closer look at the detection results. The results of one of the best-performing subjects (patient 6) are illustrated in Figure 3. From the confusion matrix we can see that our approach achieves a sensitivity of 98.4% and a specificity of 100%.

## 5. Discussion

To analyze the effectiveness of the proposed method, first, we removed the feature separation network while leaving the other settings unchanged. Then we tested on the TUH dataset using leave-one-out cross-validation. The results of the tests are shown in Table 2:

**TABLE 2:** The result after feature separation network is removed.

Methods	ACC (%)	SN (%)	SP (%)
Ours (feature separation network is removed)	81.6(±2.9)	80.5(±3.8)	82.8(±2.5)
Ours	85.7(±4.6)	83.1(±3.3)	87.2(±4.9)

By comparison, the average accuracy of the comparison method in which the feature separation network is removed is 81.6%. The proposed method ensures feature separability and improves feature discrimination, thus improving detection performance.

Second, for the invariant feature extraction network, since DANN only aligns the marginal distribution features of multi-patients, and MADA only aligns the conditional distribution features of multi-patients, we propose the method which aligns the marginal distribution and conditional distribution of each patient’s features at the same time. As the label of each training set, $y_{i}^{k}$ (*k* = 1,2) in the MADA method is modified with the value of the original label by the one-hot encoder. Then, the model is trained in the adversarial network, respectively, so that the invariant features of each category can be obtained.

To compare the advantages of the proposed method, this paper trains and tests networks that only use DANN and only use MADA. By comparing with the proposed method, the proposed method has the best performance. The results of performance comparison are shown in Table 3.

**TABLE 3:** Comparison of results with DANN and MADA methods.

Methods	ACC (%)	SN (%)	SP (%)
Only DANN (Ganin et al., 2016)	74.8(±5.3)	78.2(±6.6)	72.8(±4.1)
Only MADA (Pei et al., 2018)	79.6(±6.5)	81.5(±5.4)	77.3(±6.9)
Ours	85.7(±4.6)	83.1(±3.3)	87.2(±4.9)

**FIGURE 4:**
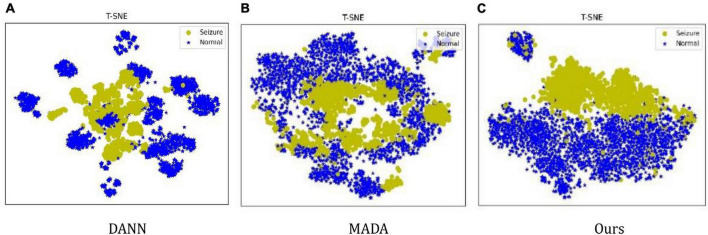
The t-SNE visualization of network feature. **(A)** Only DANN, **(B)** only MADA, and **(C)** ours.

For a clear illustration, we further use the t-SNE method (Maaten and Hinton, 2008) to visualize the feature distribution of the comparison methods, the feature distribution is illustrated in Figure 4. It can be seen that DANN only tries to align the marginal distribution. Still, due to the shift in data distribution between patients, it is difficult to align the marginal distribution, resulting in features in a decentralized state. MADA uses the aligned conditional distribution and different features are mixed together. In the proposed method, the features are clustered by category and can be discriminated. It is shown that the proposed method has advantages in learning invariant features.

The reasons are as follows: first, DANN, which uses global domain adversarial method aligns the marginal distribution of features not according to the data category; second, the MADA, which uses local domain adversarial method aligns the conditional distribution of features according to the data category; but $y_{i}^{k}$ (*k* = 1,2) in the MADA method are not the true category information, which is the output of the classification network; therefore, the features of each category cannot be aligned accurately. The proposed method uses the marginal distribution and conditional distribution alignment simultaneously, and uses the accurate label of the training set as $y_{i}^{k}$ (*k* = 1,2), which improves the performance of data feature alignment. Therefore, the proposed method has the best performance.

For future work, I suggest the following three points:

First, in the proposed method, the data features are divided into category-related features and patient-related features. In future work, the features can be divided into more detailed features, and new network structures and loss functions can be used for feature extraction to improve the algorithm’s performance.

Second, the proposed method uses adversarial training to learn the invariant features, but the results of adversarial training are not stable; there are significant differences between each training epoch; therefore, new invariant feature learning methods can be studied in the future to improve the stability of training.

Thirdly, the experiments of the proposed method are all conducted on the existing public dataset and not verified on the real clinical dataset, therefore, we need to cooperate with the clinical hospital to obtain the clinical data of epilepsy and verify the actual effect.

## 6. Conclusion

In the proposed method, a domain generalization model based on feature separation and adversarial training is proposed for the case where there is a significant shift in the data distribution between patients in the epilepsy dataset. The model includes a feature extraction network, a feature separation network, and an invariant feature extraction network. The multi-level temporal-spectral feature extraction network extracts valuable features using a convolutional operation and attention mechanism. The feature separation network is used to improve feature discrimination. The invariant feature extraction network is used to align the marginal distribution and conditional distribution of features to make the features more discriminable and general. We use the TUH dataset of 14 patients and leave-one-out cross-validation, and compared with the related literature, the proposed method achieves the best result; therefore, the proposed method can provide some reference for the clinical application of epilepsy detection.

## Data availability statement

The datasets presented in this study can be found in online repositories. The names of the repository/repositories and accession number(s) can be found in the article/supplementary material.

## Ethics statement

Ethical review and approval was not required for the study on human participants in accordance with the local legislation and institutional requirements. Written informed consent to participate in this study was provided by the participants’ legal guardian/next of kin.

## Author contributions

YY and FL conceptualized the study. YY and XQ performed the methodology. YY and HW accounted the software. YY and XL wrote and prepared the original draft. XQ and DH reviewed and edited the written draft. All authors have read and agreed to the published version of the manuscript.

## References

[B1] Ansari A. Q. Sharma P. Tripathi M. (2021). A patient-independent classification system for onset detection of seizures. *Biomed. Tech. (Berl).* 66 267–274. 10.1515/bmt-2020-0250 33548164

[B2] Azimi S. M. Fischer P. Körner M. Reinartz P. (2019). Aerial LaneNet: Lane-marking semantic segmentation in aerial imagery using wavelet-enhanced cost-sensitive symmetric fully convolutional neural networks. *IEEE Trans. Geosci. Remote Sens.* 57 2920–2938. 10.1109/TGRS.2018.2878510

[B3] Babb T. L. Wilson C. L. Isokawa-Akesson M. (1987). Firing patterns of human limbic neurons during stereoencephalography (SEEG) and clinical temporal lobe seizures. *Electroencephalogr. Clin. Neurophysiol.* 66 467–482. 10.1016/0013-4694(87)90093-9 2438112

[B4] Bui M. H. Tran T. Tran A. T. Phung D. (2021). Exploiting domain-specific features to enhance domain generalization. *NeurIPS [Preprint].* 10.48550/arXiv.2110.09410

[B5] Dissanayake T. Fernando T. Denman S. Sridharan S. Fookes C. (2021). Deep learning for patient-independent epileptic seizure prediction using scalp EEG signals. *IEEE Sensors J.* 21 9377–9388. 10.1109/JSEN.2021.3057076 34314363

[B6] Fergus P. Hignett D. Hussain A. Al-Jumeily D. Abdel-Aziz K. (2015). Automatic epileptic seizure detection using scalp EEG and advanced artificial intelligence techniques. *Biomed. Res. Int.* 2015:986736. 10.1155/2015/986736 25710040 PMC4325968

[B7] Fisher R. S. Cross J. H. French J. A. Higurashi N. Hirsch E. Jansen F. E. (2017). Operational classification of seizure types by the international league against epilepsy: Position paper of the ILAE commission for classification and terminology. *Epilepsia* 58 522–530. 10.1111/epi.13670 28276060

[B8] Ganin Y. Ustinova E. Ajakan H. Germain P. Larochelle H. Laviolette F. (2016). Domain-adversarial training of neural networks. *J. Mach. Learn. Res.* 17 2096–2030. 10.48550/arXiv.1505.07818

[B9] Hu J. Shen L. Sun G. (2018). “Squeeze-and-excitation networks,” in *Proceedings of the 2018 IEEE/CVF Conference on Computer Vision and Pattern Recognition*, Salt Lake City, UT, 7132–7141. 10.1109/CVPR.2018.00745

[B10] Indiradevi K. P. Elias E. Sathidevi P. S. Dinesh Nayak S. Radhakrishnan K. (2008). A multi-level wavelet approach for automatic detection of epileptic spikes in the electroencephalogram. *Comp. Biol. Med.* 38 805–816. 10.1016/j.compbiomed.2008.04.010 18550047

[B11] Kiral I. Roy S. Nurse E. Mashford B. Karoly P. Carroll T. (2018). Epileptic seizure prediction using big data and deep learning: Toward a mobile system. *EBioMedicine* 27 103–111. 10.1016/j.ebiom.2017.11.032 29262989 PMC5828366

[B12] Kwan P. Brodie M. J. (2000). Early identification of refractory epilepsy. *N. Engl. J. Med.* 342 314–319. 10.1056/NEJM200002033420503 10660394

[B13] Li Y. Liu Y. Cui W. G. Guo Y. Z. Huang H. Hu Z. Y. (2020). Epileptic seizure detection in EEG signals using a unified temporal-spectral squeeze-and-excitation network. *IEEE Trans. Neural Syst. Rehabil. Eng.* 28 782–794. 10.1109/TNSRE.2020.2973434 32078551

[B14] Li Y. Zheng W. Zong Y. Cui Z. Zhang T. Zhou X. (2021). A Bi-hemisphere domain adversarial neural network model for EEG emotion recognition. *IEEE Trans. Affect. Comp.* 12 494–504. 10.1109/TAFFC.2018.2885474

[B15] Liu G. Tian L. Zhou W. (2022). Patient-independent seizure detection based on channel-perturbation convolutional neural network and bidirectional long short-term memory. *Int. J. Neural Syst.* 32:2150051. 10.1142/S0129065721500519 34781854

[B16] Maaten L. Hinton G. (2008). Visualizing data using t-SNE. *J. Mach. Learn. Res.* 9 2579–2605. 10.48550/arXiv.2108.01301

[B17] Matsuura T. Harada T. (2020). Domain generalization using a mixture of multiple latent domains. *AAAI* 34 11749–11756. 10.1609/AAAI.V34I07.6846

[B18] Obeid I. Picone J. (2016). The temple university hospital EEG data corpus. *Front. Neurosci.* 10:196. 10.3389/fnins.2016.00196 27242402 PMC4865520

[B19] Pei Z. Cao Z. Long M. Wang J. (2018). “Multi-adversarial domain adaptation,” in *Thirty-Second Proceedings of the AAAI Conference on Artificial Intelligence (AAAI)*, Palo Alto, CA. 10.1609/aaai.v32i1.11767

[B20] Rahim S. S. Palade V. Jayne C. Holzinger A. Shuttleworth J. (2015). “Detection of diabetic retinopathy and maculopathy in eye fundus images using fuzzy image processing,” in *Brain Informatics and Health. BIH 2015. Lecture Notes in Computer Science, vol 9250*, eds Guo Y. Friston K. Aldo F. Hill S. Peng H. (Cham: Springer). 10.1007/978-3-319-23344-4_37

[B21] Rahim S. S. Palade V. Shuttleworth J. Jayne C. (2016). Automatic screening and classification of diabetic retinopathy and maculopathy using fuzzy image processing. *Brain Inf.* 3 249–267. 10.1007/s40708-016-0045-3 27747815 PMC5106407

[B22] Rakhade S. N. Jensen F. E. (2009). Epileptogenesis in the immature brain: Emerging mechanisms. *Nat. Rev. Neurol. J.* 5 380–391. 10.1038/nrneurol.2009.80 19578345 PMC2822660

[B23] Rasheed K. Qayyum A. Qadir J. Sivathamboo S. Kwan L. Kuhlmann P. (2021). Machine learning for predicting epileptic seizures using EEG signals: A review. *IEEE Rev. Biomed. Eng.* 14 139–155. 10.1109/RBME.2020.3008792 32746369

[B24] Schirrmeister R. Gemein L. Eggensperger K. Hutter F. Ball T. (2017). “Deep learning with convolutional neural networks for decoding and visualization of EEG pathology,” in *Proceedings of the 2017 IEEE Signal Processing in Medicine and Biology Symposium (SPMB)*, Philadelphia, PA. 10.1109/SPMB.2017.8257015

[B25] Sridevi V. Ramasubba Reddy M. Srinivasan K. Radhakrishnan K. Rathore C. Nayak D. S. (2019). Improved patient-independent system for detection of electrical onset of seizures. *J. Clin. Neurophysiol.* 36 14–24. 10.1097/WNP.0000000000000533 30383718 PMC6314507

[B26] Tang X. Zhang X. (2020). Conditional adversarial domain adaptation neural network for motor imagery EEG Decoding. *Entropy* 22:96. 10.3390/e22010096 33285871 PMC7516530

[B27] Versaci M. Morabito F. C. (2021). Image edge detection: A new approach based on fuzzy entropy and fuzzy divergence. *Int. J. Fuzzy Syst.* 23 918–936. 10.1007/s40815-020-01030-5

[B28] Versaci M. Angiulli G. Crucitti P. De Carlo D. Laganà F. Pellicanò D. (2022). A fuzzy similarity-based approach to classify numerically simulated and experimentally detected carbon fiber-reinforced polymer plate defects. *Sensors (Basel)* 22:4232. 10.3390/s22114232 35684853 PMC9185562

[B29] Waheed S. R. Rahim M. S. M. Suaib N. M. Salim A. A. (2023). CNN deep learning-based image to vector depiction. *Multimed. Tools Appl.* 82 20283–20302. 10.1007/s11042-023-14434-w

[B30] Wen Y. Zhang K. Li Z. Qiao Y. (2016). “A discriminative feature learning approach for deep face recognition,” in *Computer Vision – ECCV 2016. ECCV 2016. Lecture Notes in Computer Science, vol 9911*, eds Leibe B. Matas J. Sebe N. Welling M. (Cham: Springer). 10.1007/978-3-319-46478-7_31

[B31] White G. Cabrera C. Palade A. Li F. Clarke S. (2020). WasteNet: Waste classification at the edge for smart bins. *ArXiv [Preprint]*. 10.48550/arXiv.2006.05873

[B32] Yang Y. Qin X. L. Wen H. Li F. Lin X. G. (2023). Patient-specific approach using data fusion and adversarial training for epileptic seizure prediction. *Front. Comput. Neurosci.* 17:1172987. 10.3389/fncom.2023.1172987 37216065 PMC10192566

[B33] Yu C. Wang J. Chen Y. Huang M. (2019). “Transfer learning with dynamic adversarial adaptation network,” in *Proceedings of the 2019 IEEE International Conference on Data Mining (ICDM)*, Beijing. 10.48550/arXiv.1909.08184

[B34] Zabihi M. Kiranyaz S. Ince T. Gabbouj M. (2013). “Patient-specific epileptic seizure detection in long-term EEG recording in paediatric patients with intractable seizures,” in *Proceedings of the IET Intelligent Signal Processing Conference 2013 (ISP 2013)*, London. 10.1049/cp.2013.2060

[B35] Zhang X. Yao L. Dong M. Liu Z. Zhang Y. Li Y. (2020). Adversarial representation learning for robust patient-independent epileptic seizure detection. *IEEE J. Biomed. Health Inform.* 24 2852–2859. 10.1109/JBHI.2020.2971610 32071011

[B36] Zhao H. Zheng Q. Ma K. Li H. Zheng Y. (2021). Deep representation-based domain adaptation for nonstationary EEG classification. *IEEE Trans. Neural Netw. Learn. Syst.* 32 535–545. 10.1109/TNNLS.2020.3010780 32745012

